# Influence of rapid eye movement sleep on all-cause mortality: a community-based cohort study

**DOI:** 10.18632/aging.101858

**Published:** 2019-03-13

**Authors:** Jingjing Zhang, Xuting Jin, Ruohan Li, Ya Gao, Jiamei Li, Gang Wang

**Affiliations:** 1 Department of Critical Care Medicine, the Second Affiliated Hospital of Xi’an Jiaotong University, 710004 Xi’an, China; * Equal contribution

**Keywords:** polysomnography, rapid eye movement sleep, all-cause mortality, community

## Abstract

Introduction: Although the proportion and duration of rapid eye movement (REM) sleep are correlated with neurological and cardiovascular diseases, whether REM sleep is associated with all-cause mortality in community-based populations remains unknown.

Methods: A prospective study was performed within the Sleep Heart Health Study (SHHS, Registration NO. NCT00005275). Total sleep time, sleep efficiency, and REM sleep were measured using polysomnography. Cox proportional hazards regression models were used to estimate the association of the REM sleep with all-cause mortality.

Results: Over a mean follow-up period of 11.0 ± 3.1 y, 1234 individuals (21.9%) died. In the entire population, reduced REM sleep was significantly associated with increasing all-cause mortality. After adjustment for age, sex, race, body mass index, smoking status, total cholesterol, triglycerides, high-density lipoprotein, history of diabetes and hypertension, and the apnea–hypopnea index, the duration and proportion of REM sleep were found to be significantly associated with all-cause mortality when the lowest and the highest REM quartile groups were compared (hazard ratio, 95% confidence interval: 1.727, 1.434-2.079; 1.545, 1.298-1.839; respectively).

Conclusion: The proportion and duration of REM sleep are negatively associated with all-cause mortality. This finding emphasizes the importance of personalized sleep management in community-based populations.

## Introduction

The sleep process can be divided into two predominant phases, non-rapid eye movement (NREM) sleep and rapid eye movement (REM) sleep. The former consists of three stages (N1, N2, N3) and constitutes 75% to 80% of the total sleep [1] while the latter [2] accounts for approximately 20% to 25% of the total sleep. The REM sleep is characterized by a low-voltage, high-frequency pattern in the beta or theta range on an electroencephalograph (EEG), as well as by muscle-tone attenuation and REM [1]. Resembling wakefulness, REM sleep is a highly active mental state associated with dreaming, suspended thermoregulation, and autonomic irregularities [3]. Further, REM sleep plays a profound role in the early development of sensory systems (i.e., visual, auditory, etc.) [4], as well as learning and memory functions by selectively maintaining the newly formed synapses [5].

Over the past decade, evidences for an association between REM sleep and neurological and cardiovascular diseases have been accumulated [6,7]. Moreover, prior investigations have shown that a smaller proportion of REM sleep is associated with elevated sleep/wake blood pressure ratios [8], which may increase the risk for cardiovascular morbidity and mortality [9,10]. A longer REM stage has also been reported to hasten recovery following stroke [11]. Despite the accrual of knowledge on the effects of REM sleep, its influence on all-cause mortality remains unknown, especially in the community population. The present study, therefore investigated the association between REM sleep and all-cause mortality using data from the Sleep Heart Health Study (SHHS), a large community-based perspective cohort.

## RESULTS

### Individual selection and clinical characteristics

**Figure 1:**
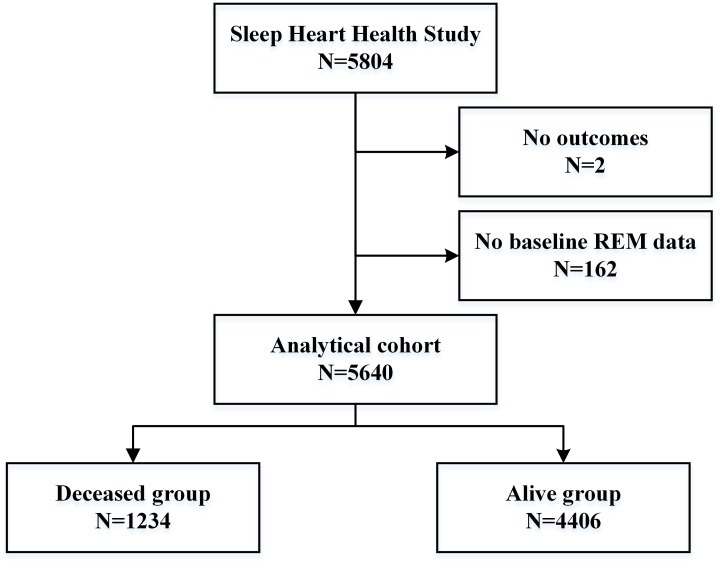
**Flow chart of participant selection.** A total of 5640 individuals were included in the analysis.

Of the 5804 participants, two had no outcomes and 162 had no baseline REM data, ultimately leaving an analytical sample size of 5640. Participants were divided into the deceased group (1234, 21.9%) and the alive group (4406, 77.1%) based on occurrence of death over a mean follow-up period of 11.0 ± 3.1 y (Fig. 1). In the present study, the longest and shortest follow-up time durations were 15.8 years and 3.7 years, respectively. Most of the study population was Caucasian (84.8%). The average age, body mass index (BMI), and apnea-hypopnea index (AHI) of the study participants were 63.14 ± 11.22 years, 28.15 ± 5.07 kg/m^2^, and 10.12 ± 13.41, respectively, in the entire population. Compared to the participants in the alive group, those in the deceased group were significantly more likely to be older (72.59 ± 9.31 vs. 60.39 ± 10.20 years) and men (53.9% vs. 46.0%) (P < 0.001 for both). In comparison to the alive group, the deceased group showed a significantly higher AHI (12.41 ± 15.33 vs. 9.53 ± 13.00 events/h), and prevalence of hypertension and diabetes mellitus; they also showed a greater decrease in total-sleep time (340.21±67.28 vs. 361.40±59.86), sleep efficiency (78.79±11.31 vs. 82.45±9.90), and amount and proportion of deep sleep (P < 0.001 for all) (Table 1).

**Table 1:** Demographic and clinical characteristics at baseline.

	**Entire population**	**Deceased**	**Alive**	***P* value**
**Subject, n**	5640	1234	4406	
**Age,yr**	63.14±11.22	72.59±9.31	60.39±10.20	<0.001
**Male, n (%)**	2691 (47.7)	665 (53.9)	2026 (46.0)	<0.001
**Race, n (%)**				<0.001
**White**	4785 (84.8)	1083 (87.8)	3702 (84.0)	
**Black**	478 (8.5)	123 (10.0)	355 (8.1)	
**Other**	377 (6.7)	28 (2.3)	349 (7.9)	
**Smoking status, n (%)**				<0.001
**Never**	2649 (47.3)	519 (42.2)	2130 (48.7)	
**Current**	537 (9.6)	107 (8.7)	430 (9.8)	
**Former**	2414 (43.1)	604 (49.1)	1810 (41.4)	
**BMI, kg/m^2^**	28.15±5.07	27.63±4.98	28.30±5.09	0.760
**AHI, events/h**	10.12±13.41	12.41±15.33	9.53±13.00	<0.001
**Hypertension, n (%)**	2390 (42.4)	750 (60.8)	1640 (37.2)	<0.001
**Diabetes, n (%)**	384 (7.1)	168 (14.0)	216 (5.2)	<0.001
**TC, mg/dL**	207.48±38.42	205.93±41.60	207.92±37.48	0.009
**HDL, mg/dL**	50.63±15.72	50.47±15.57	50.67±15.77	0.643
**Triglycerides, mg/dL**	150.26±101.76	154.97±106.76	148.97±100.32	0.677
**Sleep time (min)**	356.72±62.20	340.21±67.28	361.40±59.86	<0.001
**sleep efficiency (%)**	81.65±10.33	78.79±11.31	82.45±9.90	<0.001
**timest1p (%)**	5.44±3.95	6.09±4.63	5.26±3.72	<0.001
**timest2p (%)**	56.54±11.72	57.68±12.86	56.22±11.36	<0.001
**times3p (%)**	18.21±11.87	18.00±13.15	18.28±11.50	<0.001
**REMP (%)**	19.80±6.27	18.23±6.71	20.24±6.07	<0.001
**REM (min)**	72.36±27.49	63.53±26.98	74.84±27.13	0.932
**Deep sleep (min)**	137.93±53.53	125.22±55.22	141.49±52.50	0.037
**Deep sleep (%)**	38.02±12.78	36.23±14.11	38.52±12.34	<0.001
**ai_rem (events/h)**	15.45±10.95	14.72±11.32	15.66±10.83	0.127
**sao2 rem (%)**	94.33±2.63	93.49±2.92	94.57±2.49	<0.001
**Follow-up time (d)**	4029.47±1138.75	2586.57±1219.32	4433.59±705.19	<0.001

BMI, body mass index; AHI, apnea-hypopnea index; TC, total cholesterol; HDL, high-density lipoprotein; REM, minutes spent in rapid eye movement sleep; REMP, percent of sleep time in REM sleep; timest1p, percent of sleep time in stage 1; timest2p, percent of sleep time in stage 2; timest3p, percent of sleep time in stage 3; ai_rem, REM Arousal Index, total arousals in REM sleep per hours of REM sleep; sao2_rem, average SaO2 in REM sleep.

### Association between sleep structure and all-cause mortality

Table 2 shows the relationship between different stages of sleep and all-cause mortality. The proportions of the N1 and N2 stages were found to be positively associated with all-cause mortality in the entire population (hazard ratio [HR], 95% confidence interval [CI]: 1.045, 1.033-1.058; 1.010, 1.005-1.015; respectively). However, the relationship between the proportion of the N3 stage and all-cause mortality was non-significant. The duration of REM sleep was found to be negatively associated with all-cause mortality (HR, 95% CI: 0.987, 0.985-0.989); this association was significant after adjustments for age, gender, and race in Model 1 (HR, 95% CI: 0.990, 0.988-0.992). Though the adjustments for BMI, smoking status, total cholesterol, triglycerides, high-density lipoprotein, history of diabetes, history of hypertension, and AHI in Model 2 weakened the relationship between REM-sleep duration and all-cause mortality, the correlation remained statistically significant (HR, 95% CI: 0.992, 0.989-0.994). Similarly, the proportion of REM sleep was also negatively associated with all-cause mortality for the entire population (HR, 95% CI: 0.957, 0.948-0.965). It remained statistically significant even after adjustment for all variables (HR, 95% CI: 0.972, 0.963-0.981).

**Table 2:** Hazard ratio (95% CI) for the association between sleep architecture and all-cause mortality.

	**Univariate Model**	**Multivariate Model 1**	**Multivariate Model 2**
**Sleep time (min)**	0.995(0.994-0.996)	0.997(0.996-0.998)	0.998(0.997-0.999)
**Sleep efficiency (%)**	0.973(0.968-0.979)	0.983(0.976-0.989)	0.984(0.977-0.991)
**timest1p (%)**	1.045(1.033-1.058)	1.027(1.014-1.040)	1.019(1.005-1.033)
**timest2p (%)**	1.010(1.005-1.015)	1.005(1.000-1.010)	1.003(0.997-1.008)
**times3p (%)**	0.997(0.992-1.002)	1.002(0.997-1.007)	1.003(0.998-1.009)
**REMP (%)**	0.957(0.948-0.965)	0.965(0.956-0.974)	0.972(0.963-0.981)
**REM (min)**	0.987(0.985-0.989)	0.990(0.988-0.992)	0.992(0.989-0.994)
**Deep sleep (min)**	0.995(0.993-0.996)	0.996(0.995-0.998)	0.998(0.996-0.999)
**Deep sleep (%)**	0.987(0.982-0.991)	0.992(0.988-0.997)	0.995(0.990-1.000)
**ai_rem (events/h)**	0.992(0.986-0.997)	0.989(0.984-0.995)	0.985(0.979-0.992)
**sao2 rem (%)**	0.928(0.919-0.938)	0.945(0.934-0.957)	0.940(0.925-0.955)

Model 1: adjusted for age (category), gender, race; Model 2: Model 1 plus body mass index, smoking status, total cholesterol, triglycerides, high-density lipoprotein, history of diabetes, and history of hypertension, apnea-hypopnea index.

REM, minutes spent in rapid eye movement sleep; REMP, percent of sleep time in REM sleep; timest1p, percent of sleep time in stage 1; timest2p, percent of sleep time in stage 2; timest3p, percent of sleep time in stage 3; ai_rem, REM Arousal Index, total arousals in REM sleep per hours of REM sleep; sao2_rem, average SaO2 in REM sleep.

### Association between REM sleep and all-cause mortality

To further explore the relationship between REM sleep and all-cause mortality, we divided the population into quartiles on the basis of the amount of REM sleep. Using the highest quartile as a reference, the HR of all-cause mortality in the quartile with the lowest REM-sleep duration was 2.474 (95% CI, 2.087-2.932). However, after adjustment for age, sex, and race in Model 1, the influence of REM sleep duration on all-cause mortality was slightly diminished (HR, 95% CI: 1.933, 1.630-2.294). More adjustment with BMI, AHI, smoking status, blood lipid spectrum, and histories of diabetes and hypertension in Model 2, led to a further decrease in the intensity of the association, which nevertheless remained statistically significant (HR, 95% CI: 1.727, 1.434–2.079). In addition, we also found a linear association between REM sleep-duration quartiles and all-cause mortality (P for trend < 0.001 in both Model 1 and Model 2). Similarly, with the highest quartile as a reference, the association between the lowest proportion of REM sleep and all-cause mortality was significant after adjustment for all variables in the regression model (HR, 95% CI: 1.545, 1.298-1.839), and the linear association also existed between REM-sleep proportion quartiles and all-cause mortality (P for trend < 0.001) (Table 3).

**Table 3:** Hazard ratio (95% CI) for the association between REM sleep quartiles and all-cause mortality.

		**Quartiles of rapid eye movements**				**Overall Tendency**
	**Q1 (low)**		**Q2**	**Q3**	**Q4 (high)**	
**REM (min)**						
**Subjects, n**	1438		1384	1434	1384	
**Events, n (%)**	449 (31.2)		321 (23.2)	275 (19.2)	189 (13.7)	
**Univariate Model**	2.474 (2.087–2.932)		1.774 (1.483–2.124)	1.451 (1.206–1.746)	1 (Ref)	0.748(0.711-0.788)
**Model 1**	1.933 (1.630–2.294)		1.440 (1.202–1.724)	1.248 (1.037–1.503)	1 (Ref)	0.805(0.764-0.848)
**Model 2**	1.727 (1.434–2.079)		1.384 (1.141–1.680)	1.202 (0.986–1.466)	1 (Ref)	0.835(0.789-0.884)
**REMP (%)**						
**Subjects, n**	1411		1407	1415	1407	
**Events, n (%)**	429 (30.4)		306 (21.7)	273 (19.3)	226 (16.1)	
**Univariate Model**	2.020 (1.720–2.373)		1.385 (1.166–1.644)	1.219 (1.022–1.453)	1 (Ref)	0.793(0.753-0.884)
**Model 1**	1.715 (1.460–2.016)		1.256 (1.057–1.491)	1.150 (0.964–1.372)	1 (Ref)	0.836(0.759-0.880)
**Model 2**	1.545 (1.298–1.839)		1.257 (1.046–1.510)	1.129 (0.934–1.364)	1 (Ref)	0.865(0.819-0.914)

Model 1: adjusted for age (category), gender, race; Model 2: Model 1 plus body mass index, smoking status, total cholesterol, triglycerides, high-density lipoprotein, history of diabetes, and history of hypertension, apnea-hypopnea index.

REM, minutes spent in rapid eye movement sleep; REMP, percent of sleep time in REM sleep.

## DISCUSSION

Our study identified an association between the duration and proportion of REM sleep and all-cause mortality. Moreover, we found that all-cause mortality decreases with an increase in the duration and proportion of REM sleep. This association was found to be independent of age, sex, race, BMI, AHI, smoking status, blood lipid spectrum, history of diabetes and hypertension. In addition, we found a positive correlation between all-cause mortality and the proportion of N1 and N2 stages (light sleep). However, our study did not identify a statistically significant association between the proportion of N3 stage and all-cause mortality, likely because the N3 stage alone has no obvious predictive value in the community population.

Sleep is indispensable to not only the central nervous system but also to all the other physiological systems [1]. Previous studies have linked the quality of sleep with sleep architecture, which is composed of NREM sleep and REM sleep. The former can be further divided into three stages: N1, N2, and N3. The NREM sleep maintains cell homeostasis by reducing the number of synaptic connections to a basic level, which is necessary for normal physical and intellectual performance and behavior [12]. Along with the REM sleep, the N3 stage, defined as deep sleep, reflects the sleep quality [6]. Prior research has identified relationships between the REM-sleep and neurological and cardiovascular diseases, patients with cognitive impairment exhibit reduced REM sleep [13,14]; Pase et al. found that relatively shorter REM sleep is associated with a higher risk of developing dementia [15]. Giubilei et al. found that the number and duration of REM phases were significantly reduced in the acute phase of ischemic stroke as well as correlated with the severity of neurological deficits at prognosis [16]. Further, an observational study of 186 middle-aged African-American and Caucasian adults revealed that the greater sleep/wake ratios of blood pressure were correlated with a smaller proportion of REM sleep [8], which may increase the risk of cardiovascular morbidity and mortality [9,10]. However, to the best of our knowledge, this study is the first to evaluate the association between the amount of REM sleep and all-cause mortality.

Recent investigations have suggested that the initiation and maintenance of REM sleep is due to certain brainstem structures [11,17,18]. Glutamatergic neurons in the sublaterodorsal tegmental (SLD) nucleus function as REM-on neurons while GABAergic neurons situated in the pons function as REM-off neurons during NREM sleep [19,20]. Furthermore, another study found that REM sleep is triggered when the REM-off neurons are inactivated by the REM-on GABAergic neurons localized in the ventrolateral periaqueductal gray and the dorsal paragigantocellular nucleus [21]. The disinhibited ascending SLD REM-on neurons then induce cortical activation, while the descending ones initiate muscle atonia and sensory inhibition [22]. The triggering of REM sleep may be explained by the recruitment of neurons in the suprachiasmatic nucleus (SCN), which underlie the circadian clock critical for establishing the circadian rhythm in animals [23]. Moreover, multiple investigations have indicated the correlation between neuronal electrical activity originating in the SCN and the sleep stages [24,25].

Considering the association between SCN and sleep architecture, we speculate that aberrant SCN activity may underlie the shortening of REM sleep. In addition to the disruptions of circadian rhythms concomitant with aging [26], research has shown that the amount of REM sleep is reduced by approximately 50% in late life [27,28]. Further, both the circadian rhythm and the length of REM sleep are affected by psychosocial stress and functioning of the hypothalamus-pituitary-adrenal (HPA) axis, a system important for the regulation of stress responses. Several studies have reported that dysregulation of the HPA axis leads to higher evening cortisol concentrations and shorter REM-sleep duration [27,29]. Furthermore, multiple investigations have revealed that disruption of the circadian rhythm in critically ill patients in the intensive care unit may be explained by reduced REM sleep [30]. Decreased REM sleep may therefore indicate an aberrant circadian rhythm, and hence, a decreased SCN function. The disruptions to daily sleep/wake rhythms as well as many physiological and neuroendocrine cycles may ultimately account for a rise in the risk of all-cause mortality.

This study is subject to several limitations. First, the present study was mainly composed of middle-aged and older Caucasian adults, and therefore may not be generalized to all age and ethnic groups. Second, as the causes of death in the SHHS were not obtained, the relationship between REM sleep and specific fatal diseases should be explored by future analyses. Third, only the baseline measurement of REM sleep monitored by in-home polysomnography was available for the individuals in this dataset. In future, long-term REM sleep monitoring should be performed to clarify the association between REM sleep and all-cause mortality. Lastly, we did not further analyze the effect of REM sleep waveform on all-cause mortality due to pilot study. The microstructure of REM stage and detailed recordings of specific waveform across subjects by means of EEG are required for further investigation. Notwithstanding these limitations, the results presented here help to fill an important gap in the existing literature regarding the association between REM sleep and all-cause mortality.

In conclusion, increased sleep time and amount of REM sleep are associated with less all-cause mortality in the studied cohort of middle-aged and older adults. Our findings provide further evidence that the duration and proportion of REM sleep may serve as predictive factors for all-cause mortality. Investigation into the relationship between REM sleep and specific fatal diseases is therefore warranted. Further, as sleep architecture is regulated by circadian rhythm, we hypothesize that the extent of REM sleep may reflect the SCN functioning.

## METHODS

### Study design and participants

The SHHS (registration number, NCT00005275) is a community-based, multi-center, prospective cohort study designed to identify the cardiovascular outcomes of sleep-disordered breathing among individuals aged ≥ 40 years. Of the 11145 people who were eligible for enrollment in the SHHS from 1995-1998, 5804 ultimately agreed and provided informed consent to complete the baseline interview and health examination. The heart-health outcomes including survival or decease were monitored until 2011. The protocol was approved by the Institutional Review Board of each participating institution: Boston University, Case Western Reserve University, Johns Hopkins University, Missouri Breaks Research Inc., New York University Medical Center, University of Arizona, University of California at Davis, University of Minnesota-Clinical and Translational Science Institute, and the University of Washington. The data were accessed on the basis of a signed agreement with the Brigham and Women’s Hospital, Boston, MA, USA [31].

### Polysomnography

Participants in the SHHS underwent an in-home EEG-based polysomnography performed with the Compumedics P-series portable monitor (Abbotsford, Victoria, Australia). Details regarding the polysomnographic equipment, hook-up procedures, scoring, quality assurance and control have been described elsewhere [32]. Sleep recordings were staged in epochs of 30 s and categorized as N1 (stage 1 sleep), N2 (stage 2 sleep), N3 (stage 3 sleep), REM (REM sleep), or W (awake); the first two stages constitute light sleep, while N3 and REM comprise deep sleep [33]. All other sleep parameters were calculated on the basis of these sleep stages. Total-sleep time was obtained by measuring the time spent across all sleep stages; sleep efficiency was obtained by comparing the time spent asleep relative to that spent in bed.

### Assessment of mortality

When subjects could not be contacted for their scheduled follow-up, every attempt was made to determine whether or not they were deceased. All the known contacts for the subject were called to determine the subject’s vital status. Moreover, both the local death registries and the National Death Index were searched for the subject’s name or social security number. When a death was ascertained, the parent study obtained records from any hospitalization that occurred within one month of death, a copy of the death certificate, and, if performed, an autopsy report. In addition, the subject’s physician and his or her family member or other proxy who was with the subject when he or she passed away was interviewed to obtain details concerning the circumstances of the death.

### Covariates

During the SHHS home visit, a study technician collected each participant’s health history using a standardized questionnaire before polysomnogram monitoring was performed. Smoking status was classified as ‘never’ (if the participant reported smoking fewer than 20 packs of cigarettes during his or her lifetime), ‘former’, or ‘current’. Participants’ BMI and blood-lipid measurements were obtained using standardized protocols at the SHHS baseline examination. History of hypertension was identified based on the second and third blood pressure readings or if the participant was being treated with hypertension agents. History of diabetes mellitus was ascertained based on the report of a physician-administered diagnosis or on the reported use of insulin or oral hypoglycemic medication. The AHI was defined as the average number of apneas and hypopneas, each associated with a 4% decrease in oxygen saturation per hour of sleep [31].

### Statistics analyses

Values represent the mean ± standard deviation for continuous variables and percentages for categorical variables. Cox proportional hazards regression modelling was used to examine the association between REM sleep and all-cause mortality. Multivariate models were adjusted for the following factors: (i) age, gender and race in Model 1; (ii) age, gender, race, BMI, smoking status, total cholesterol, triglycerides, high-density lipoprotein, history of diabetes mellitus and history of hypertension and AHI in Model 2. P values of < 0.05 were considered to be statistically significant. All statistical analyses were performed with the SPSS software package version 22.0 (IBM Corp., Armonk, NY, USA).
